# Dengue Virus Envelope Protein Domain III Induces Nlrp3 Inflammasome-Dependent NETosis-Mediated Inflammation in Mice

**DOI:** 10.3389/fimmu.2021.618577

**Published:** 2021-03-17

**Authors:** Te-Sheng Lien, Der-Shan Sun, Shih-Che Hung, Wen-Sheng Wu, Hsin-Hou Chang

**Affiliations:** ^1^Department of Molecular Biology and Human Genetics, Tzu-Chi University, Hualien, Taiwan; ^2^Institute of Medical Sciences, Tzu-Chi University, Hualien, Taiwan; ^3^Division of General Surgery, Department of Surgery, Hualien Tzu Chi Hospital, Buddhist Tzu Chi Medical Foundation, Hualien, Taiwan; ^4^School of Medicine, Tzu Chi University, Hualien, Taiwan

**Keywords:** dengue envelope protein domain III, dengue hemorrhage fever, neutrophil, neutrophil extracellular traps, NEtosis, Nlrp3 inflammasome, pyroptosis

## Abstract

Abnormal immune responses and cytokine storm are involved in the development of severe dengue, a life-threatening disease with high mortality. Dengue virus-induced neutrophil NETosis response is associated with cytokine storm; while the role of viral factors on the elicitation of excessive inflammation mains unclear. Here we found that treatments of dengue virus envelope protein domain III (EIII), cellular binding moiety of virion, is sufficient to induce neutrophil NETosis processes *in vitro* and *in vivo*. Challenges of EIII in inflammasome *Nlrp3*^−/−^ and *Casp1*^−/−^ mutant mice resulted in less inflammation and NETosis responses, as compared to the wild type controls. Blockages of EIII-neutrophil interaction using cell-binding competitive inhibitor or selective Nlrp3 inflammasome inhibitors OLT1177 and Z-WHED-FMK can suppress EIII-induced NETosis response. These results collectively suggest that Nlrp3 inflammsome is a molecular target for treating dengue-elicited inflammatory pathogenesis.

## Introduction

Dengue is one the most important mosquito borne diseases in the tropical and subtropical areas of the world (1, 2), while specific treatments and effective vaccines are currently unavailable (3–8). Infections with dengue viruses (DENV) can lead to a wide range of clinical manifestations and disease severity. Severe dengue (also known as dengue hemorrhage fever, DHF) is characterized by plasma leakage and abnormal bleeding that can lead to shock and high mortality. Because DHF typically occurs during secondary infections with DENVs, abnormal adaptive immune responses are considered as part of the pathophysiology. For example, reports have suggested that antibody-dependent enhancement (9), original antigenic sin (10), autoantibody production (11) may be involved. However, detrimental innate immune responses such as excessive inflammation and cytokine storm are likely the critical pathological changes that lead to exacerbated disease, tissue injuries and ultimate death in DHF (9, 10, 12–15).

The mechanism underlying dengue-induced unregulated inflammation remains elusive (9, 10, 12, 13). In the innate immune system, neutrophils are first line of defense against infection through engulfment of microbes, secretion of anti-microbials and induction of neutrophil extracellular traps (NETs)-releasing cell death process termed NETosis (16, 17). NETs are extracellular DNA-protein complexed networks, which bind pathogens and modulate inflammation (16, 18). Pathogenic roles of NETosis have been found in non-infectious diseases, such as autoimmunity, coagulation, acute injuries and cancer (19). In addition, NETosis has been reported associating with cytokine storm in various infectious diseases, including dengue (14, 20, 21). Interleukin (IL)-1β, a potent proinflammatory cytokine released by DENV-infected leukocytes, has been considered as a critical component in cytokine storm (22–24). Inflammasomes, cellular sensors for pathogen associated molecular patterns (PAMPs) and damage associated molecular patterns (DAMPs), are critical for IL-1 activation (14, 25), and the cell death process pyroptosis (26). Reports revealed that the elevated levels of circulating IL-1β and gene expression in DHF patients suggesting the involvement of IL-1β in the disease severity (27, 28). IL-1β enhances the vascular permeability, particularly in association with other proinflammatory cytokines such as tumor necrosis factor (TNF)-α and interferon in clinical profiles of DF and DHF (14, 29–31).

In our previous reports, in the two-hit model, we found that sequential injections of DENV (32) or DENV-envelope protein domain III (rEIII) (33) (1st hits) plus anti-DENV non-structural protein NS1 antibody (anti-NS1 Ig; 2nd hit) to simulate the disease progression of DHF, induced hemorrhage pathogenesis recapitulate certain disease-signatures of DHF, including thrombocytopenia, plasma leakage, vascular injury, hemorrhage, liver damage, and high mortality (32, 33). In addition to these manifestations, we also found that circulating proinflammatory cytokine levels such as TNF, IL-1, and IL-6, are greatly increased (32, 33). Intriguingly, IL-1 receptor antagonist (IL-1RA) treatments greatly ameliorated such 2-hit induced pathogenesis (32, 33). In addition, Nlrp3 deficiencies as observed in the *Nlrp3*^−/−^ and *Casp1*^−/−^ mutant mice, also greatly reduced 2-hit induced pathogenesis (32, 33). These results collectively suggested that Nlrp3 inflammasome-IL-1 axis is involved in dengue induced pathogenesis in this DENV-, and EIII-induced hemorrhage mouse models.

The DENV viral factor that contributes to NETosis remains unclear. Plasma EIII levels could be detected in acute DENV infection (34). Evidences have shown that EIII treatments induced inflammasome activation and inflammation of macrophages (35). Our previous study revealed that challenges with the DHF-viral-load-equivalent levels of EIII can suppress megakaryocyte, and endothelial cell function through initiating cell death (33, 36). Accordingly, EIII may be a cytotoxic virulence factor of DENV to cause NETosis and initiate downstream inflammation. As a result, in this present study, we would like to investigate whether DENV and EIII can directly initiate NETosis, and whether Nlrp3 inflammasome is involved. In addition, whether we can ameliorate EIII-mediated inflammation through suppression of Nlrp3 inflammasome and NETosis pathways is also addressed. Relevant implications and applications are discussed.

## Materials and Methods

### DENV, Recombinant Protein, and Antibodies

Mosquito C6/36 cell line (ATCC CRL-1660) and DENV-2 (PL046) were maintained and amplified using standard cell culture methods (36–38). Soluble recombinant proteins glutathione-S transferase (rGST), and EIII (rEIII) were obtained from cultured bacteria (*Escherichia coli*) (39), after isopropyl β-D-1-thiogalactopyranoside induction, and were purified as previously described (32, 33, 36). To reduce endotoxin (lipopolysaccharide; LPS) contamination to a desired level (<1 EU/mg protein), the lysate- and resin-packed column was washed with a buffer (8 M urea, 100 mM NaH_2_PO_4_, and 10 mM Tris-HCl; pH = 6.3) with the addition of 1% Triton X-114 (Sigma–Aldrich, St. Louis, MO, USA). The rEIII was eluted with a buffer (8 M urea, 100 mM NaH_2_PO_4_, and 10 mM Tris-HCl; pH = 4.5) and refolded using a linear 4–0 M urea gradient in a dialysis buffer (2 mM reduced glutathione, 0.2 mM oxidized glutathione, 80 mM glycine, 1 mM EDTA, 50 mM Tris-HCl, 50 mM NaCl, and 0.1 mM phenylmethylsulfonyl fluoride) at 4°C for 2–3 h, as previously described (36). The purity of the rEIII protein can reach ~90%. The LPS contamination was monitored with a Limulus Amoebocyte Lysate QCL-1000 kit (Lonza, Walkersville, MD, USA) (36, 37, 40). Batches of purified recombinant proteins with an LPS contamination level of <1 EU/mg of protein were used. The pre-immune control Ig, anti-NS1 Ig, and anti-EIII Ig from experimental rabbits (New Zealand White; *Oryctolagus cuniculus*) were obtained before and after rNS1- and rEIII-immunizations according to previously described methods (41). According to previously described methods (33), recombinant proteins (50 μg/mL) were used to block rEIII-cell (neutrophil) binding, including recombinant mouse dendritic cell-specific intercellular adhesion molecule-3-grabbing non-integrin (DC-SIGN; CD209), DC-SIGNR, C-type lectin domain family 5 member A (CLEC5A) and CLEC2 (R&D Systems, Indianapolis, IN, USA). To analyze the binding properties of rEIII proteins on protein-coated beads (latex, 1.1 μm, Sigma-Aldrich) and mouse neutrophils, rEIII protein were conjugated with biotin by using an EZ-Link™ Sulfo-NHS-Biotinylation kit (Thermo Fisher Scientific). The rEIII-beads binding experiments were performed using biotin-labeled rEIII proteins (300 μg/mL, 20 μL) incubated with protein-precoated beads [2 μg protein coating with 1 mg/mL beads 1 h in total 50 μL phosphate buffered saline PBS, blocking with 1% bovine serum albumin (BSA, Sigma-Aldrich)/PBS, 30 min, resuspended in 20 μL PBS after wash]. The levels of biotin-labeled rEIII proteins bound to beads or neutrophils [50 μg/mL competing protein + (2 × 10^5^) cells/mL in culture medium for 30 min] were determined through flow cytometry by PE/Cy5 avidin (Biolegend, San Diego, CA, USA) staining. DENV-2 envelope protein fragment (32 kDa, domain I + domain II; ProSpec-Tany TechnoGene, Ness-Ziona, Israel) was used as a control protein. The rEIII-competitive inhibitor chondroitin sulfate B (CSB, 10 μg/mL; Sigma-Aldrich) was used to suppress rEIII-induced neutrophil binding and cell death. Anti-citrullinated histone H3 (CitH3; citrulline R8), anti-histone H2A family member X (H2AX), and anti-gasdermin D (GSDMD) antibodies (Abcam, Cambridge, UK) were used for flow cytometry NETosis analysis.

### Experimental Mice

Wild-type mice of ages 8–12 weeks in C57BL/6J background were purchased from the National Laboratory Animal Center (Taipei, Taiwan) (38, 42–46). Gene knockout mice with a C57BL/6J background, including *Nlrp3*^−/−^ and *Casp1*^−/−^ (32), were obtained from the Center National de Recherche Scientifique (Orléans, France) (32, 33, 47). All experimental animals were housed in the Animal Center of Tzu-Chi University in a specific-pathogen-free, temperature-, and lighting-controlled environment with free access to filtered water and food. All genetic knockout strains were backcrossed with the wild-type C57Bl/6J mice for at least 6 generations. After challenged with vehicle (saline), BSA (a control protein, 2 mg/kg), DENV (1.2 × 10^7^ PFU /kg; DHF viral load) and rEIII (2 mg/kg; a dosage equivalent to 1.2 × 10^7^ PFU/kg), experimental mice were immediately rescued with or without Nlrp3 inhibitor OLT1177 treatments (50 mg/kg). Plasma levels of IL-1β, TNF-α, and CitH3 of the experimental mice were determined through enzyme-linked immunosorbent assay (ELISA) (IL-1β, TNF-α, Biolegend; CitH3, Cayman Chemical, Ann Arbor, MI, USA) 1 d after rEIII treatments; neutrophils were isolated and analyzed (see following “Analyses of neutrophils”).

### Ethics Statement

The animal experiments in this report were conducted in agreement with National (Taiwan Animal Protection Act, 2008) directive for protection of laboratory animals. All experimental protocols for examining the experimental animals were approved by the Animal Care and Use Committee of Tzu-Chi University, Hualien, Taiwan (approval ID: 101019).

### Analyses of Neutrophils

Blood samples of mice were collected via the retro-orbital venous plexus using plain capillary tubes (Thermo Fisher Scientific, Waltham, MA, USA), and then transferred into polypropylene tubes (Eppendorf; Thermo Fisher Scientific) containing anticoagulant acid-citrate-dextrose solution (ACD; 38 mM citric acid, 75 mM sodium citrate, 100 mM dextrose) (48, 49). Following previously described methods (50), mouse neutrophils were purified from mouse blood samples using Ficoll-Paque (Ficoll-Paque Plus, 1.077 g/mL, GE Healthcare, Chicago, IL, USA) and dextran (Sigma-Aldrich) sedimentation (3% w/v) density gradient centrifugation and red blood cell lysis. To obtain fluorescent NET images, mouse neutrophils (1 × 10^5^) were treated with vehicle (the diluent, normal saline, 0.9 % NaCl), rEIII (50 μg/mL or an equivalent dose 0.6 μM), DENV (1 × 10^5^ PFU/mL, an equivalent dose of rEIII is 0.6 μM) or 12-O-tetradecanoylphorbol-13-acetate (TPA, 2 nM, Sigma-Aldrich) for 2 h at 37C. After fixation by 4% paraformaldehyde on coverslips, these neutrophils were then stained with rabbit anti-mouse citrulline Histone H3 antibody (1:1000) and 4',6-diamidino-2-phenylindole (DAPI, 5 μl/ml). A fluorescence microscope (Nikon Eclipse E800; Nikon Taiwan, Taipei, Taiwan) (51) was used for obtaining the NETosis images. To analyze mitochondria membrane potential, superoxide, and membrane potential levels, mitochondria labeling reagents MitoTracker™ Green FM (Thermo Fisher Scientific, Waltham, MA, USA), MitoSOX™ Red mitochondrial superoxide indicator (Thermo Fisher Scientific), MitoTracker™ Red CMXRos (Thermo Fisher Scientific) were used according to the manufacturer's instructions (33, 52). A flow cytometer (FACSCalibur; BD Biosciences, San Jose, CA, USA) (36, 38) was used in this study to analyze RCD, cell live/death and mitochondria metabolic activities with or without rEIII challenges and RCD or signaling pathway (e.g., PAD4, Nlrp3 inflammasome) inhibitor treatments (33). Levels of cellular reactive oxygen species (ROS) were analyzed using 2′,7′-dichlorofluorescin diacetate (Sigma-Aldrich) staining-flow cytometry analysis.

### Analyses of Regulated Cell Death

To analyze DENV or rEIII induced neutrophil cell death, mouse neutrophils were incubated with DENV or rEIII for 1 h and then subjected to flow cytometry analyses after washed with PBS. Various regulated cell death (RCD) responses, including apoptosis (CaspGLOWTM Red Active Caspase-3 Staining Kit, #K193, BioVision, Milpitas, CA, USA), autophagy (Cyto-ID™ Autophagy Detection Kit, Enzo Life Sciences, #ENZ51031, Farmingdale, NY, USA), ferroptosis (C11 BODIPY 581/591, #27086, Cayman Chemical), necroptosis (RIP3/B-2 alexa Fluor 488, Santa Cruz Biotechnology, #sc-374639 AF488, Santa Cruz, CA, USA), pyroptosis (Caspase-1 Assay, Green, #9146, ImmunoChemistry Technologies, MI, USA), and live/dead cell labeling (Zombie NIR™ Fixable Viability Kit, #423105, Biolegend), were analyzed using respective cell labeling reagents (30 min in PBS). Treatments (1 h) of cell death inducers were used as positive controls for various type of regulated cell death (RCD; apoptosis: doxorubicin, 2.5 μg/mL, Nang Kuang Pharmaceutical, Taipei, Taiwan; autophagy: rapamycin, 250 nM, #R0395, Sigma-Aldrich; ferroptosis: erastin, 10 μM, #17754, Cayman Chemical; necroptosis, TNF-α, 2 ng/mL, #575202, Biolegend; pyroptosis: nigericin, 3.5 μM, #6698, ImmunoChemistry Technologies, Minnesota, USA; NETosis, TPA, #P8139, 2 nM Sigma-Aldrich) (30 min in PBS). Inhibitors were used to address the involvements of specific RCD pathways (apoptosis: Z-DEVD-FMK, 10 μM, #FMK004, R&D Systems, Indianapolis, IN, USA; autophagy: Chloroquine diphosphate, 60 μM, #C6628, Sigma-Aldrich; ferroptosis: Ferrostatin-1 2.5 μM, #17729, Cayman Chemical; necroptosis: Necrostatin-1, 50μM, #11658, Cayman Chemical; pyroptosis: Z-WHED-FMK 10 μM, #FMK002, R&D Systems; pyroptosis/GSDMD: dimethyl fumarate (DMF), 25–100 μM, #14714, Sigma–Aldrich; NETosis: peptidyl arginine deiminase 4 (PAD4) inhibitor GSK484, 10 μM, #17488, Sigma–Aldrich; Nlrp3: OLT1177, 10 μM, #24671, Cayman Chemical; EIII competitive blocker: Chondroitin sulfate B, CSB, 10 μg/mL, #C3788, Sigma–Aldrich; 30 min pretreatments before addition of DENV, rEIII and cell-death inducers according to the manufacturer's instructions).

### Statistical Analyses

In this report, the means, standard deviations, and statistics of the quantifiable data were calculated using the SigmaPlot 10 and SPSS 17 software packages. Significance of the data was examined using one-way ANOVA, followed by the *post hoc* Bonferroni-corrected *t*-test. The probability of type-1 error α = 0.05 was recognized as the threshold of statistical significance.

## Results

### Nlrp3 Inflammasome Is Involved in rEIII Induced Neutrophil NETosis

**Figure 1:**
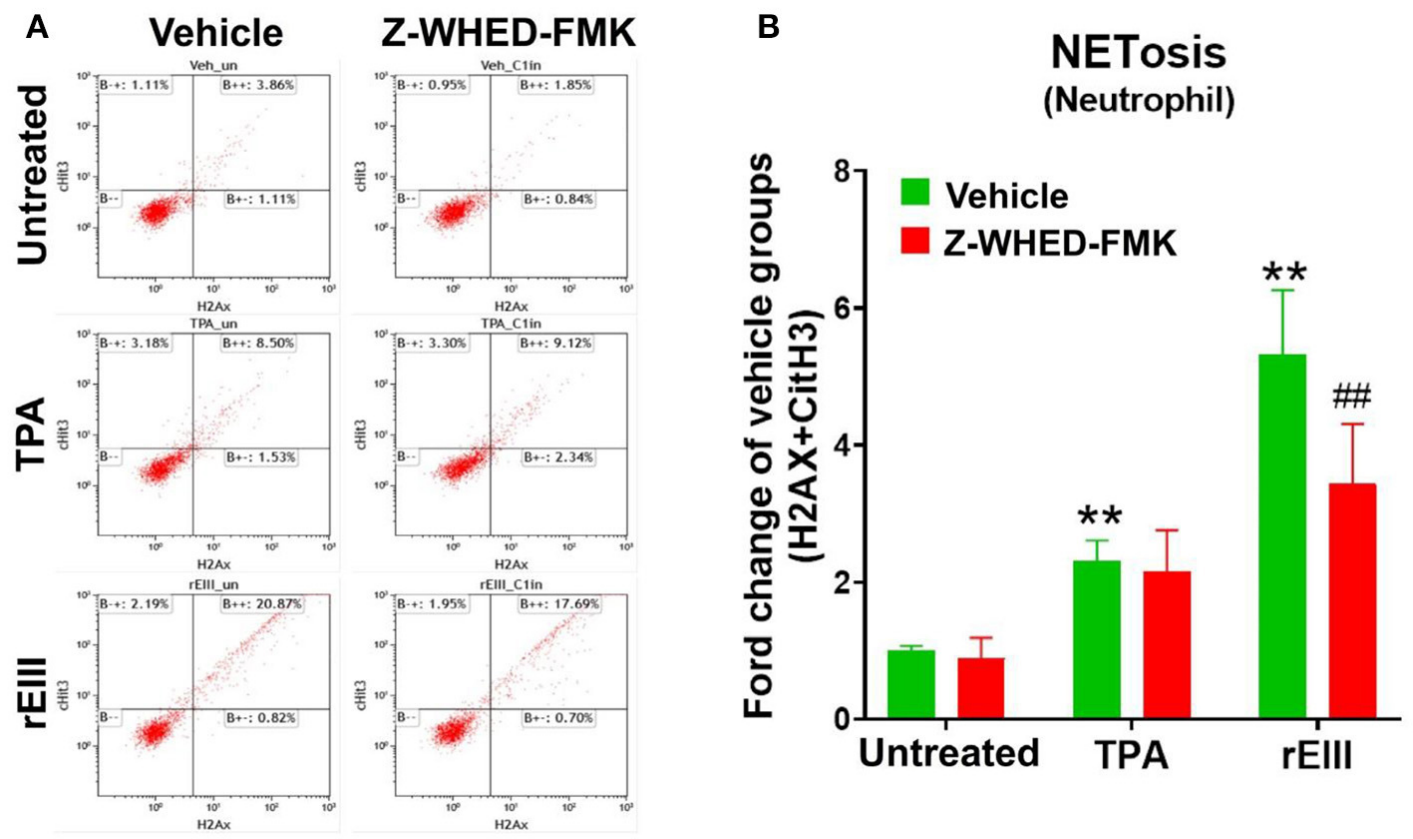
Essential role of caspase 1 in DENV rEIII-induced NETosis. The gating of flow cytometry analysis of vehicle, 12-O-tetradecanoylphorbol-13-acetate (TPA, a positive control NETosis inducer; 2 nM) and DENV rEIII (0.6 μM) challenged (1 h) wild mice neutrophils (1 × 10^5^) with or without caspase 1 inhibitor Z-WHED-FMK pretreatments (30 min) **(A)**. The quantified results of flow cytometry analysis, which reveal that TPA and rEIII treatments markedly induced NETosis formation; by contrast, caspase 1 inhibitor Z-WHED-FMK treatments can only considerably suppress rEIII-induced NETosis, but not TPA-induced NETosis **(B)**. *n* = 6, **P < 0.01, vs. untreated controls; ^*##*^*P* < 0.01 vs. vehicle groups.

**Figure 2:**
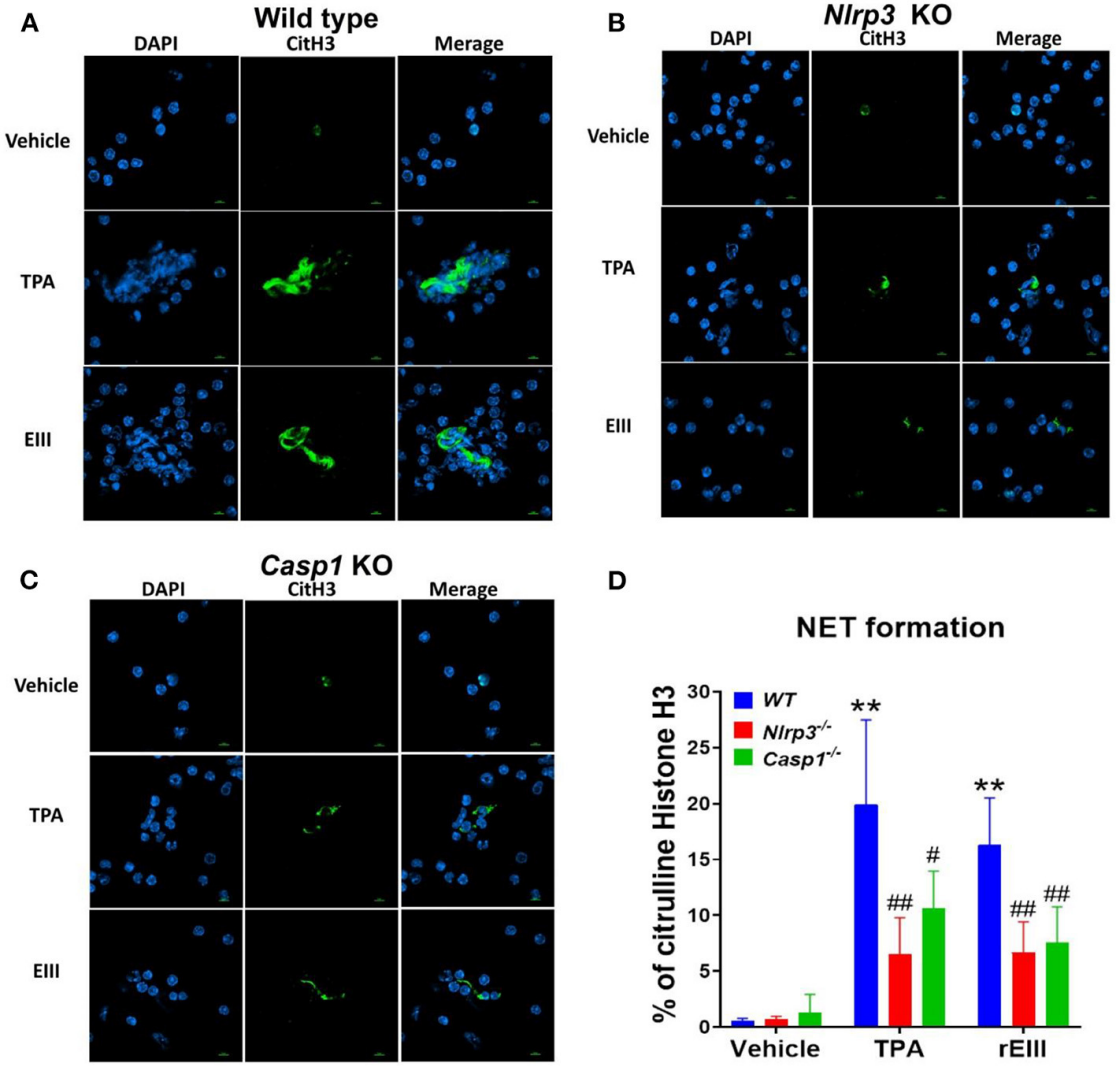
Essential role of Nlrp3 inflammasome in DENV rEIII-induced NETosis. After challenged (1 h) by TPA (2 nM) and DENV rEIII (0.6 μM), the images of DAPI (nucleus DNA staining) and citrullinated histone H3 (CitH3; NETosis marker) staining of NETosis formation of neutrophils from wild type **(A)**, Nlrp3 null (*Nlrp3*^−/−^) **(B)**, and caspase 1 null (*Casp1*^−/−^) **(C)** mouse were showed. The quantified results from flow cytometry are also indicted **(D)**. *n* = 6, ** *P* < 0.01, vs. vehicle controls; ^#^*P* < 0.05, ^*##*^*P* < 0.01 vs. wild type (WT) groups. Scale bars: 5 μm.

Flow cytometry analysis of NETosis markers citrullinated histone H3 (CitH3) (53, 54) and histone H2A family member X (H2AX) (55, 56) revealed that rEIII is sufficient to initiate NETosis in neutrophils; the potency of rEIII is comparable to the classical NETosis inducer phorbol ester (18, 57) (Figure 1A, Supplementary Figure 1, flow cytometry gating; Figure 1B). In addition, such induction of NETosis can be suppressed by treatments of inflammasome/caspase 1 inhibitor Z-WHED-FMK (Figure 1). In agreement with this, we found that, when compared to the neutrophils from wild type mice, Nlrp3 inflammasome-deficient (*Nlrp3*^−/−^ and *Casp1*^−/−^) neutrophils displayed relatively low NETosis levels after treated with rEIII and TPA (Figures 2A–C, cell images; green channels: CitH3, a NETosis marker; Figure 2D, quantified results). These data suggest that Nlrp3 inflammasome plays critical role in rEIII-induced NETosis.

### DENV and rEIII Induce Multiple Regulated Cell Death Pathways of Neutrophils

NETosis is a type of regulated cell death (RCD) (58). Previous reports suggested that neutrophil RCD exacerbate pathogenesis in infectious diseases (59, 60). As a result, we would like to investigate whether various RCD pathways are also involved in DENV- and rEIII-induced neutrophil cell death.

**Figure 3:**
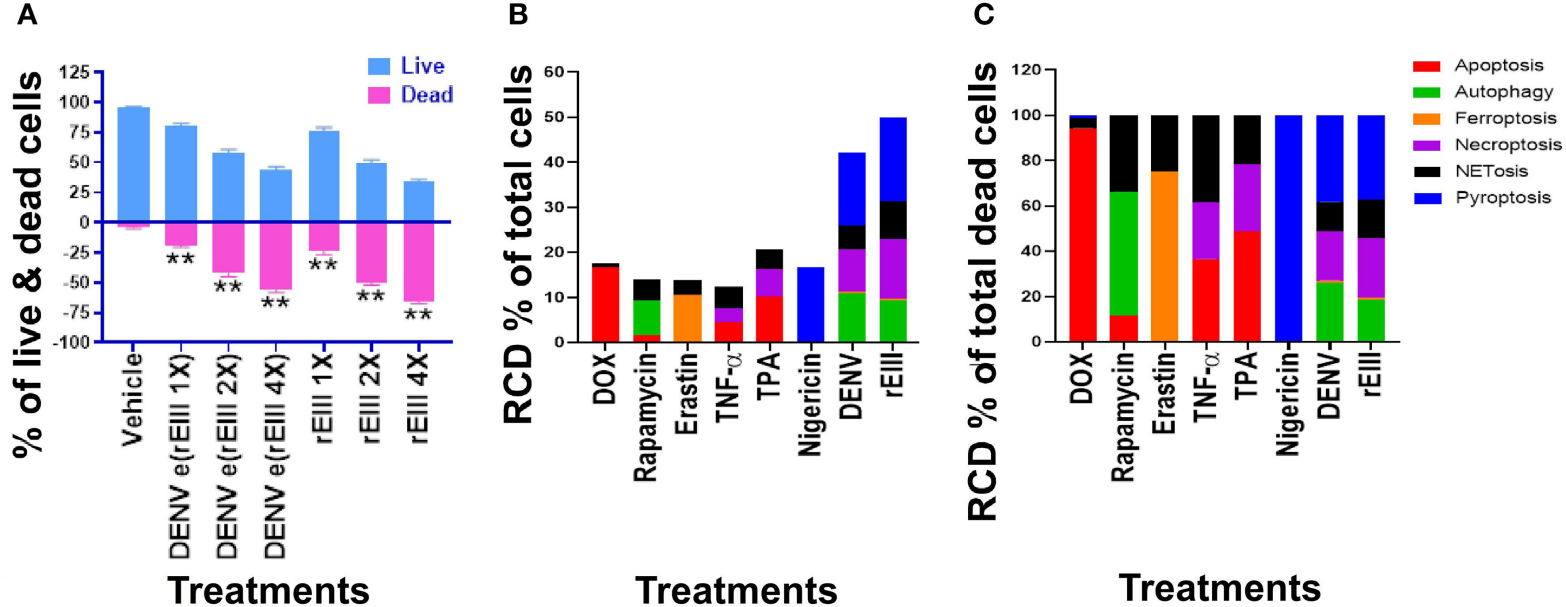
DENV- and rEIII-induced regulated cell death in neutrophils. **(A)** Wild type mouse neutrophils treated (1 h) with vehicle and various doses of DENV and rEIII; the live and death cell populations were revealed by Zombie-NIR Kit labeling and flow cytometry analysis. [rEIII 1× = 0.3 μM, 2× = 0.6 μM, 4× = 1.2 μM,; DENV e(rEIII 1×) is a DENV level equivalent to 0.3 μM rEIII, as indicted by the methods described elsewhere (33)]. **(B)** Treatments (1 h) of regulated cell death (RCD) inducers, doxorubicin (DOX; apoptosis) (2.5 μg/mL), rapamycin (autophagy) (0.5 μM), erastin (ferroptosis) (10 μM), TNF-α (necroptosis) (2.5 ng/mL), TPA (NETosis) (2 nM), and nigericin (pyroptosis) (3.5 μM) induced relatively simple RCD patterns. By contrast, DENV and rEIII induced multiple RCD pathways, in which pyroptosis is the major RCD response, counts ~40% of total RCD and the NETosis response displays only ~20% total RCD. **(C)** If the respective RCDs are normalized by the population of death cells (dead cell population normalized to 100%), we can obtain a more similar RCD pattern in DENV and rEIII groups (flow cytometry gating and calculation methods described in Supplementary Figure 2). DENV 1× = 4.2 × 10^4^ PFU/mL, 2× = 8.4 × 10^4^ PFU/mL, 4× = 1.7 × 10^5^ PFU/mL; **P < 0.01 vs. vehicle groups.

We first found that DENV and rEIII induced neutrophil cell death in a dose dependent manner (Figure 3A). Overall, various cell death inducers, including doxorubicin (apoptosis) (61, 62), rapamycin (autophagy) (63), erastin (ferroptosis) (64), TNF-α (necroptosis) (65, 66), nigericin (pyroptosis) (67), served as positive control agents to induce respective RCD pathways of the tested neutrophils (Figures 3B,C, dead cell population adjusted to 100%; Supplementary Figure 2, flow cytometry gating and calculation). Notably, when compared with cell death agonists, DENV and rEIII treatment induced considerable pyroptosis, necroptosis, autophagy and NETosis responses in the neutrophils, while only minor or no ferroptosis and apoptosis levels (Figure 3B, % of total cells; Figure 3C, % of total dead cell). In addition, the cell type specific RCD patterns/profiles (CTS-RCDPs) (33) of neutrophil in the DENV-, and rEIII-treated groups were somewhat similar, with pyroptosis exhibiting the highest levels in both groups among all tested RCD pathways (Figure 3B; ~40%), suggesting that DENV-induced CTS-RCDP in the neutrophils is likely mediated through EIII on the DENV virion. In case one dead cell may display multiple RCDs, here we defined CTS-RCD as a detection ratio of RCDs in 1 cell type at a specific condition.

An unexpected finding is that the DENV and rEIII induced NETosis only displayed approximately 20% of total RCDs (Figure 3B); and a classical NETosis inducer TPA (a phorbol ester) also induced NETosis about only 40% of total RCDs (Figure 3B, TPA groups). This let us wondered whether DENV and EIII-mediated induction of such a low percentage of NETosis in total RCD, could sufficiently lead to neutrophil dysfunction. In addition, we would like to investigate whether Nlrp3 inflammasome is involved in rEIII-induced neutrophil death. Accordingly, Nlrp3 inhibitor OLT1177 and inflammasome/caspase1 inhibitor Z-WHED-FMK were used to further characterizations of whether Nlrp3 inflammasome is involved in respective RCD responses.

**Figure 4:**
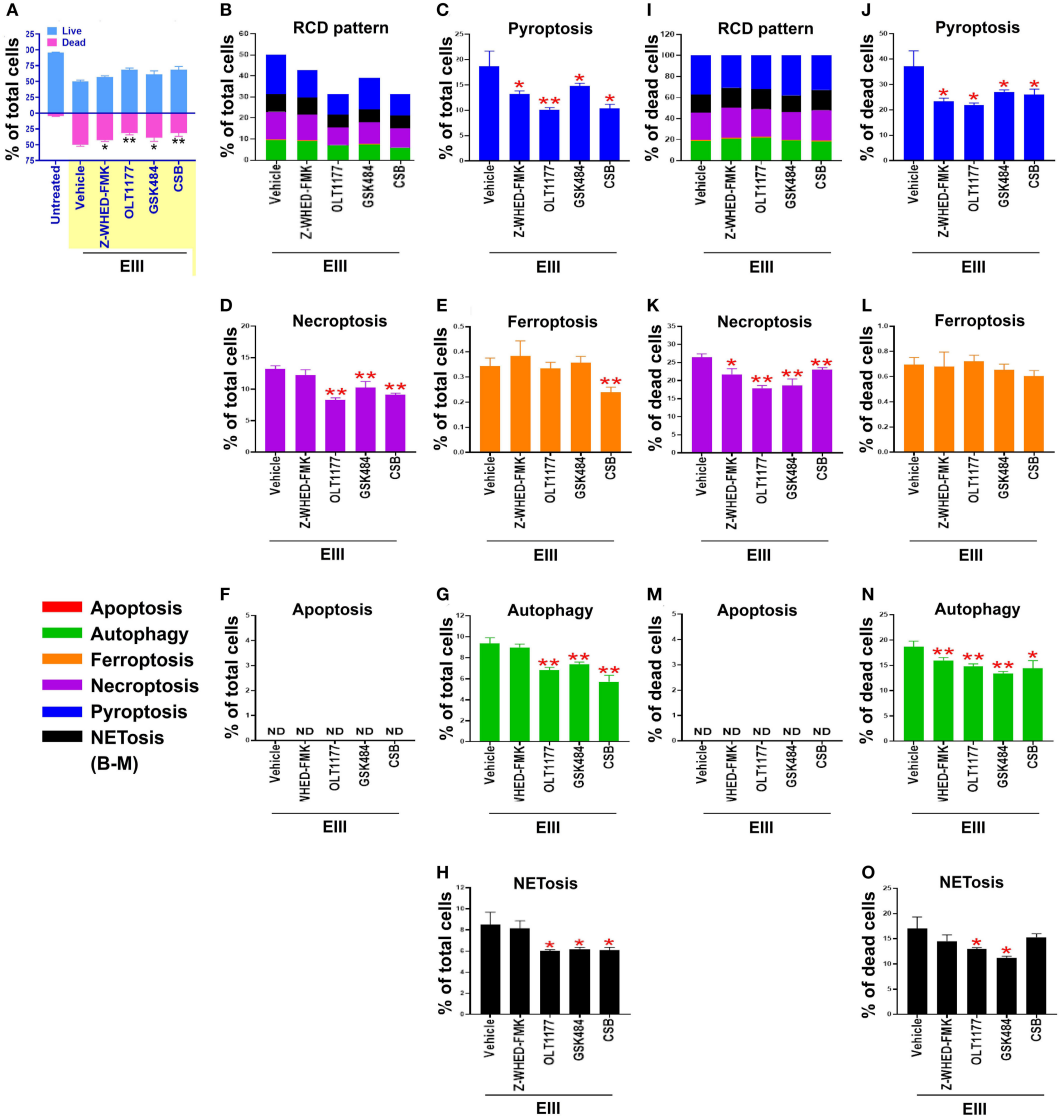
Protection of neutrophils from rEIII-induced pyroptosis under treatment with Nlrp3 inflammasome inhibitors. Pre-treatments (30 min) with NETosis inhibitor GSK484 (2 nM), Nlrp3 inhibitor OLT1177 (10 μM), and caspase 1 inhibitor Z-WHED-FMK (10 μM) on the rescue of rEIII-induced neutrophil total cell death **(A)**. Pretreatments (30 min) with Nlrp3 inflammasome inhibitors OLT1177, Z-WHED-FMK, and NETosis inhibitor GSK484 rescued rEIII-induced (1 h) neutrophil pyroptosis **(B,C)**, necroptosis **(D)**, autophagy **(G)** and NETosis **(H)**, but not ferroptosis and apoptosis **(E,F)**. If the respective RCD% was normalized by the population of death cells [**(I)**: dead cell population normalized to 100%], we found that CSB, OLT1177 and Z-WHED-FMK still display rescue effects on pyroptosis **(J)**, necroptosis **(K)**, autophagy **(N)**, and NETosis **(O)**, but not ferroptosis and apoptosis **(L,M)**. Chondroitin sulfate B (CSB, 10 μg/mL), a competitive inhibitor against rEIII binding (33), serving as a positive inhibitor control to inhibit cell death. *n* = 6, *P < 0.05, **P < 0.01, vs. respective vehicle groups. ND, not detected.

We found that treatments with inflammasome inhibitors OLT1177 and Z-WHED-FMK both suppressed EIII-induced neutrophil cell death (Figures 4A,B; Supplementary Figure 3, percentage pie charts of OLT1177 and Z-WHED-FMK treatments; equivalent to some data in Figures 4A,B,I). In addition, OLT1177 and Z-WHED-FMK suppressed pyroptosis (Figures 4C,J), necroptosis (Figures 4D,K), autophagy (Figures 4G,N), and NETosis (Figures 4H,O), with (Figures 4I–O) or without (Figures 4B–H) normalization of dead cell population. These results suggested that pyroptosis is the major RCD of rEIII-induced neutrophil death, and which can be rescued by selective inhibitors against Nlrp3 inflammasome. In addition, Nlrp3 likely involved in neutrophil NETosis as treatments of selective inhibitor OLT1177 rescued total neutrophil death (Figure 4A) and NETosis (Figures 4H,O). Consistently, treatments of pyroptosis/GSDMD inhibitor DMF (68) suppressed EIII-induced neutrophil pyroptosis, NETosis, cellular ROS, surface GSDMD levels (Supplementary Figure 4, flow cytometry analyses), and caspase-1 activation (Supplementary Figure 5, colorimetric assay). Furthermore, despite NETosis displayed only ~20% cell death (Figures 4B,I), PAD4 inhibitor GSK484 suppressed considerable levels of total neutrophil death (Figure 4A) and RCDs including pyroptosis, necroptosis, autophagy, and NETosis (Figure 4). These evidences collectively suggesting that that RCDs pyroptosis, necroptosis, and autophagy are likely associated to NETosis, and NETosis is still a critical RCD pathway of neutrophil in response to rEIII exposure.

### Nlrp3 Inflammasome Deficiency and Inhibitor Treatments Rescue DENV- and rEIII- Exacerbated Mitochondria Metabolic Burden and Inflammation of Neutrophils

**Figure 5:**
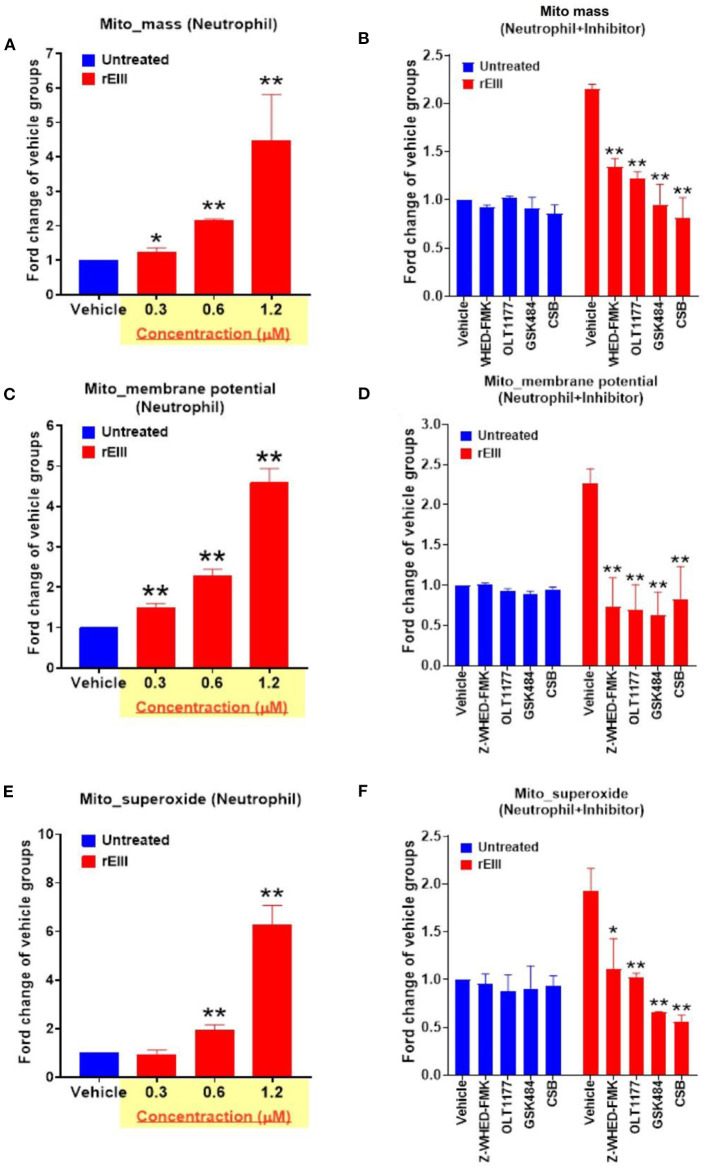
DENV rEIII-induced mitochondria ROS production is ameliorated by Nlrp3 inflammasome and NETosis inhibitor. The rEIII (0.6 μM)-induced (1 h) elevations of neutrophil mitochondria mass **(A)** membrane potential **(C)** and superoxide **(E)** levels in a dose dependent manner. The induction of these mitochondria metabolic burdens in the neutrophils could be suppressed by treatments of Nlrp3 and caspase 1 inhibitors Z-WHED-FMK (10 μM) and OLT1177 (10 μM), NETosis inhibitor GSK484 (10 μM), and the cell-binding competitive inhibitor chondroitin sulfate B (CSB, 10 μg/mL), respectively **(B,D,F)**. *n* = 6, *P < 0.05, **P < 0.01, vs. respective untreated vehicle groups **(A,C,E)**; *P < 0.05, **P < 0.01, vs. respective vehicle groups **(B,D,F)**.

**Figure 6:**
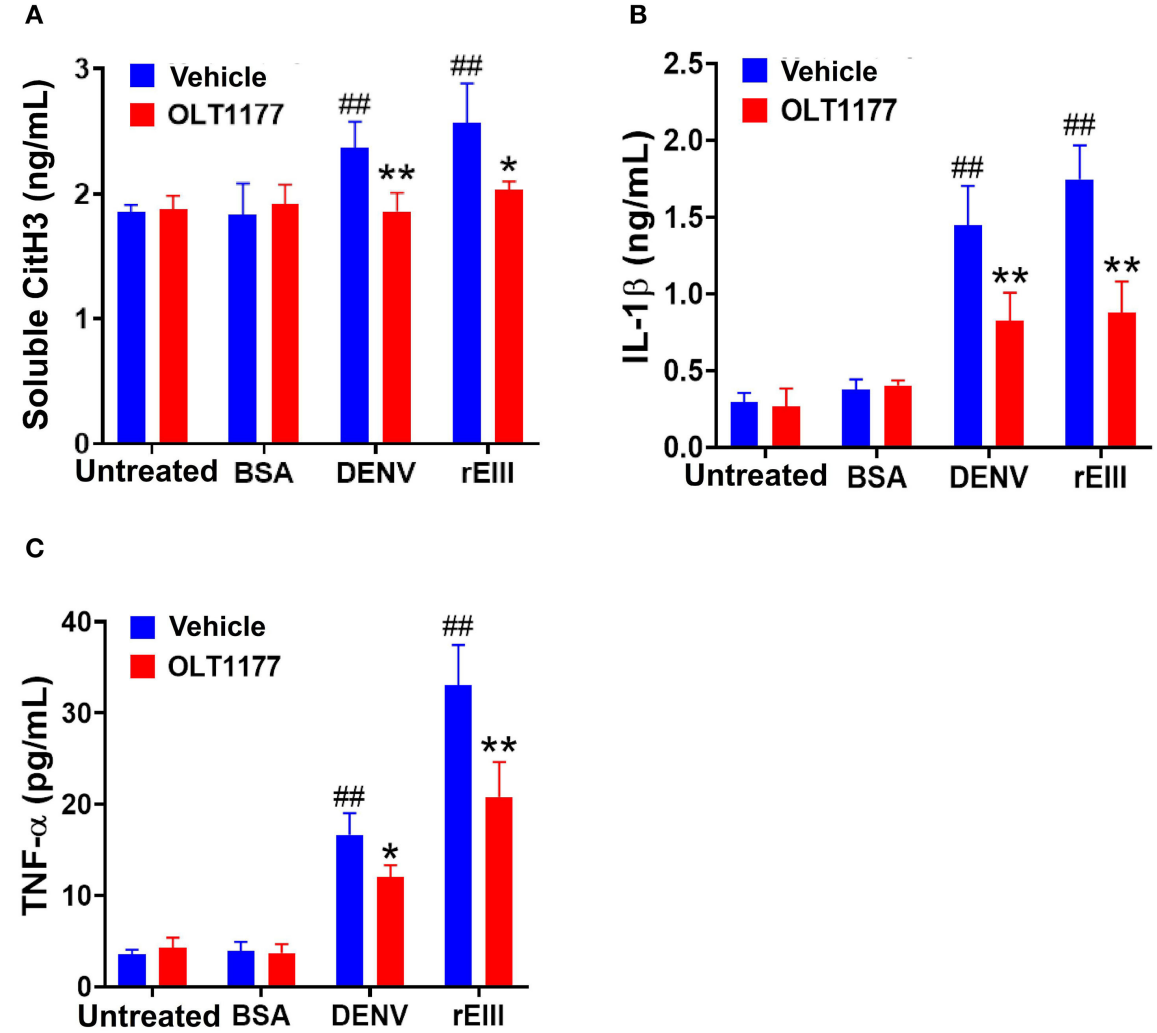
Nlrp3 inhibitor OLT1177 protects mice from DENV- and rEIII-induced NETosis and IL-1β, TNF-α production in mice. After challenged with vehicle (saline), BSA (a control protein) 2 mg/kg), DENV (1.2 × 10^7^ PFU /kg; DHF viral load) and rEIII (2 mg/kg; a dosage equivalent to DENV 1.2 × 10^7^ PFU/kg), experimental mice were immediately rescued with or without Nlrp3 inhibitor OLT1177 treatments (50 mg/kg). The ELISA analyzed plasma levels of circulating soluble citrullinated histone H3 (CitH3; a NETosis marker) **(A)**, circulating IL-1β levels **(B)**, and TNF-α levels **(C)** after 1 d treatments were showed. *n* = 6, ^*##*^*P* < 0.01, vs. respective untreated groups; *P < 0.05, **P < 0.01, vs. respective vehicle groups.

**Figure 7:**
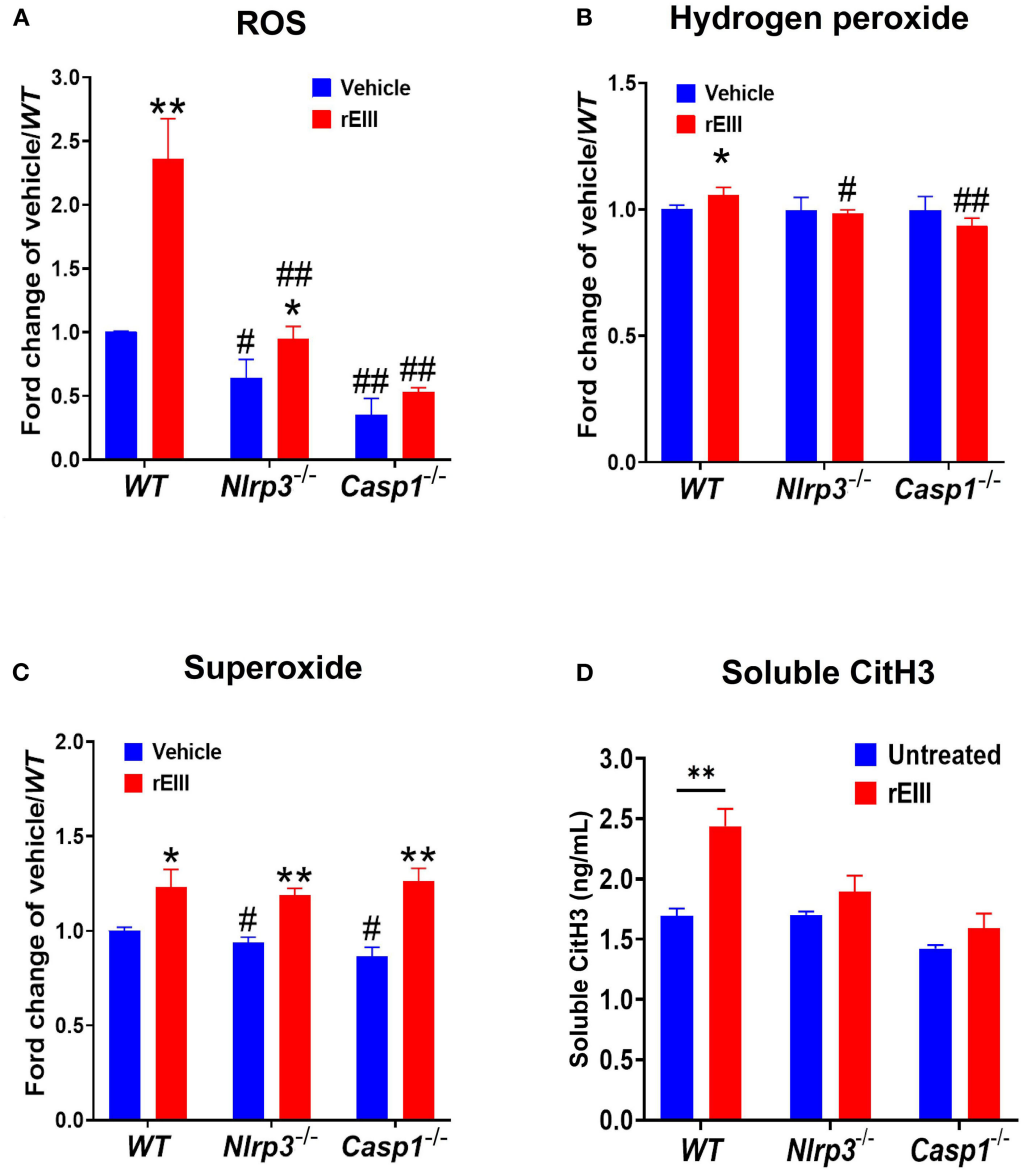
Nlrp3 and caspase 1 deficiencies protest rEIII-induced neutrophil ROS production and NETosis. Treatments (1 h) of rEIII (0.6 μM)-induced elevations of neutrophil cellular reactive oxygen species (ROS) **(A)** hydrogen peroxide **(B)**, superoxide **(C)** *in vitro*. Treatments (1 d) of rEIII (2 mg/kg)-induced elevations of circulating CitH3 **(D)** levels in wild type mice. By contrast, such rEIII (0.6 μM *in vitro*, 2 mg/kg in mice)-induced stimulations are markedly reduced in *Nlrp3*^−/−^ and *Casp1*^−/−^ gene knockout mice in both *in vitro* and *in vivo* experiments **(A–D)**. *n* = 6, ^#^*P* < 0.05, ^*##*^*P* < 0.01, vs. respective wild type (WT) groups; *P < 0.05, **P < 0.01, vs. respective vehicle groups.

Because inflammasome-mediated pyroptosis is a major RCD involving in rEIII-induced neutrophil defect, here we would like to further investigate whether suppression of neutrophil Nlrp3 inflammasome through inhibitor treatments is sufficient to ameliorate DENV rEIII-induced neutrophil defects. Here we found that treatments of rEIII-increased mitochondria mass (Figure 5A), membrane potential (Figure 5C) and superoxide (Figure 5E) levels in a dose dependent manner, while treatments of Nlrp3 inflammasome inhibitors OLT1177 and Z-WHED-FMK ameliorated such metabolic burden of neutrophil mitochondria (Figures 5B,D,F). Levels of caspase-1 activation in the neutrophils were analyzed and confirmed in parallel using colorimetric assay (Supplementary Figure 5). In agreement with this, mouse experiments further revealed that, treatments of Nlrp3 inflammasome inhibitors OLT1177 markedly ameliorated rEIII- induced elevation of circulating soluble CitH3 (Figure 6A), IL-1β (Figure 6B), and TNF-α (Figure 6C) levels in mice. Consistently, compared to wild type mice, neutrophils from *Nlrp3*^−/−^ and *Casp1*^−/−^ mutant mice displayed markedly reduced levels of total mitochondria ROS (Figure 7A), hydrogen peroxide (Figure 7B), superoxide (Figure 7C) after *in vitro* treatments of rEIII. Similarly, rEIII treatments markedly induced circulating CitH3 in wild type mice, but not in *Nlrp3*^−/−^ and *Casp1*^−/−^ mutant mice (Figure 7D). These results collective suggest that EIII is a virulence factor to induce neutrophil defects, and Nlrp3 inflammasome is a critical target for DENV and EIII to induce neutrophil dysfunction, NETosis, and inflammation.

## Discussion

Inflammasomes, cellular molecular sensors for PAMPs and DAMPs, are multimeric protein complexes comprising of NLRs [nucleotide-binding domain (NBD) and leucine-rich-repeat-(LRR)-containing], the absent in melanoma-2 (AIM2)-like receptors (ALRs), an adaptor molecule ASC (apoptosis-associated speck-like protein containing a CARD), and procaspase-1 (14, 25, 69, 70). Activation of inflammasomes leads to protein-cleavage processes, turning pro-caspase 1 into active form caspase 1, which converts pro-IL-1β and pro-IL-18 into respective active forms (IL-1β and IL-18) (69). Uncontrolled inflammasome activation exacerbates autoimmune and excessive inflammatory pathogenesis in infectious diseases, despite the adequate levels of inflammasome activation can help defending the pathogens (71). As a result, selective inhibitors against inflammasomes have been developed for treating various inflammatory and infectious diseases, and have got promising advancements (70, 72). High circulating levels of IL-1β and IL-18 have been associated DHF (27, 73), suggesting a critical role of inflammasomes in DENV-induced pathogenesis. Mouse experiments further revealed that DENV induced NET formation and inflammasome activation was impaired in neutrophils from *CLEC5A*^−/−^ and *TLR2*^−/−^ mutants (74). DENV-induced NET leads to endothelial cell damage and vascular leakage. Evidences have suggested that NET formation contributes significantly in disease pathogenesis, and NET components were more predominantly displayed in serum samples of DHF patients (75). Despite of these findings, the mechanism underlining DENV-induced NETosis formation is not fully understood; particularly, the viral factor leading to NETosis remains elusive. Here we found that, the DENV EIII could be a potential virulence fact that elicits abnormal neutrophil responses and NETosis. Because DENV and EIII induced similar RCD patterns of neutrophils (Figure 3B, DENV and rEIII groups), suggesting that, during the speak viremia stage, high levels of DENV virion conduct the cytotoxicity to enhance NETosis through EIII moiety. This is partly consistent with the findings that NET formation is markedly increased in neutrophils isolated from dengue patients during the acute phase of the infection (75). Additionally, our data suggests that such cell-type-specific RCD patterns may be a useful analysis method on the functional characterization of biologically drugs and hazardous materials at the cellular levels.

Inflammasomes regulate and interact with various RCDs (72, 76–78). The inhibitors of either Nlrp3 inflammasome/pyroptosis (inhibitor OLT1177) or NETosis (inhibitor GSK484) can suppress to each other (Figure 4), suggests there could exist a cross-talk between these 2 pathways. First, such cross-talk could be regulated at an intracellular signaling level; for example, GSDMD, an effector protein of pyroptosis, plays a critical role in the generation of NET (79). Consistently, here we found that treatments of GSDMD inhibitor DMF drastically reduced EIII-induced pyroptosis and NETosis in neutrophils (Supplementary Figure 4). Accordingly, if Nlrp3 inflammasome-activated GSDMD can further enhance NET formation, it is reasonable to observe an inhibition of NETosis using inhibitors from a pyroptosis pathway. Second, the cross-talk may also explain by a model of intercellular regulations between macrophage and neutrophil. For example, the NET is able to prime macrophages to produce IL-1 and IL-18 through the Nlrp3 inflammasome, thus amplifying the inflammatory response (80). At the same time, IL-18 effectively stimulated NET release and caspase-1 activation in primed macrophages compared to IL-18 alone (80). This suggests a feed-forward loop that NET increase the production of IL-1β and IL-18 in macrophages, which in turn can stimulate NET formation in neutrophils (80). These evidences may explain our observations that treatments of Nlrp3 inhibitor OLT1177 can markedly suppressed rEIII-induced neutrophil pyroptosis and NETosis *in vitro* (Figure 4), and markedly suppressed rEIII-induced NETosis and inflammation in mice (Figure 6). However, as these regulations still not been clearly demonstrated in dengue, more detailed mechanism is worth of further investigations.

As a glycosaminoglycan (GAG) binding lectin (81), EIII could have multiple neutrophil surface targets. Recent evidences have suggested that lectin DC-SIGN mediated DENV infection in dendritic cells (82); lectins CLEC2 and CLEC5A plays critical roles on DENV-induced inflammation (24, 74, 83, 84). CLEC5A and DC-SIGN are expressed by leukocyte subpopulations (82, 84); while their respective isoforms CLEC2 and DC-SIGNR preferentially expressed by the other cell types (84, 85). Here we used recombinant soluble CLEC2, CLEC5A, DC-SIGN, DC-SIGNR, to perform EIII competition experiments. Analysis results revealed that CLEC5A-, and DC-SIGN-, but not CLEC2-, and DC-SIGNR-coated beads can bind to rEIII (Supplementary Figure 6). Consistently, CLEC5A and DC-SIGN, but not CLEC2 and DC-SIGNR can block rEIII-neutrophil binding (Supplementary Figure 6). In addition, CLEC5A and DC-SIGN can reduce EIII-induced increased ROS and NETosis levels in neutrophils (Supplementary Figure 6). These evidences collectively suggested that CLEC5A and DC-SIGN are cellular targets of EIII on neutrophils. As a critical inflammatory regulator, the role of CLEC5A and DC-SIGN in EIII- mediated pathogenesis is worthy of further investigations.

Cell population is heterogeneous even in one cell line. This could be a reason that reports have revealed treatments of pathogens and cytotoxic agents leading to multiple types of RCDs simultaneously (86–92). For example, when cellular stress can activate both receptor-induced lysosomal-dependent, and mitochondrial-mediated cell death pathways, which will lead to both programmed necrosis and apoptosis (88). Similarly, as DENV and EIII been reported to have multiple cellular targets, it is reasonable to detect multiple RCDs after DENV and EIII challenges. Here, we found that DENV and EIII but not the other RCD inducers, induced a similar RCD pattern in neutrophils (Figures 3B,C). Previous reports have suggested that, upon stimulation by PAMP, neutrophil activation results in necrotic RCDs (93, 94); by contrast, neutrophil apoptosis contributes to the resolution of inflammation (93–96). Consistently, here we found that DENV and EIII (pathogen, PAMP) preferentially induced necrotic RCDs with almost no detectable apoptosis levels in neutrophils (Figure 4). Although further investigations are needed, our data suggested that the neutrophil CTS-RCDPs are useful on the characterization of cellular tendency on the induction of RCDs in particular conditions.

In addition to EIII, DENV membrane protein (M) and non-structural proteins NS2A, NS2B were also shown to induce inflammasome activation (14, 97, 98), and NS1 was demonstrated to enhance inflammation through a toll-like receptor 4 (99, 100). NS2A, NS2B have shown to serve as viroporins to increase cell-membrane permeability and activate inflammasome (14, 101, 102). Viroporins primarily affect virus-infected cells (103), and clinical course of DHF occurs specifically when the viremia markedly decreased (104, 105); these evidences suggest that NS2A, NS2B-mediated inflammasome activations may be not timely associated with the clinical course of DHF. DENV virus particle-expressed EIII and soluble NS1 are detected at high levels prior to the acute phase of DHF (104, 105), and may be considered as two virulence factors for severe dengue. Here we found that both EIII and NS1 treatments can induce increased NETosis levels in neutrophils, and EIII has a relative higher activity (Supplementary Figure 7; DENV envelop domain I, domain II protein fragment, served as a control protein). Because the induction of virion-expressed EIII and soluble NS1 are induced in a similar, but not a same time course (105), the respective pathogenic role of EIII and NS1 on the elicitation of DHF-related pathogenesis remains to be further studied. However, data obtained in the study suggested that virion-associated EIII is a candidate virulence factor that contributes to dengue-elicited NET formation.

In this present study, we found that treatments of both DENV and EIII, a cell-surface-binding and cytotoxic protein, can induce multiple cell death pathways in neutrophils with a similar cell-type-specific RCD pattern (Figure 3B). NETosis inhibitor GSK484 treatments sufficiently suppressed EIII-induced cell death (Figure 4A), NETosis (Figure 4G), and mitochondria metabolic burden (Figure 5) of neutrophils. This suggested that, despite the NETosis seems to be a relatively minor response that counts approximately 20% of total neutrophil RCDs, the EIII-stimulated NETosis is sufficiently leading to an abnormal neutrophil activation and cell death. In addition, our data revealed that blockage of Nlrp3 inflammasome, through treatments of inhibitor OLT1177 or gene deficiencies in Nlrp3 and caspase 1 expression, protects neutrophils from rEIII-induced NETosis (Figures 4, 7) and proinflammatory cytokines TNF and IL-1 secretion in mice (Figure 6). These results collectively suggest that Nlrp3 inflammasome is a promising target for treating DENV-induced inflammation.

## Data Availability Statement

The original contributions presented in the study are included in the article/Supplementary Material, further inquiries can be directed to the corresponding author/s.

## Ethics Statement

The animal study was reviewed and approved by Animal Care and Use Committee of Tzu-Chi University, Hualien, Taiwan (approval ID: 101019).

## Author Contributions

H-HC conceptualized and supervised this project and wrote this manuscript. T-SL, D-SS, S-CH, and W-SW performed experiments and analyzed the data. All authors contributed to the article and approved the submitted version.

## Conflict of Interest

The authors declare that the research was conducted in the absence of any commercial or financial relationships that could be construed as a potential conflict of interest.
