# Supplementary material for: Dengue Virus Envelope Protein Domain III Induces Nlrp3 Inflammasome-Dependent NETosis-Mediated Inflammation in Mice
Source: Front Immunol. 2021 Mar 17;12:618577. doi: 10.3389/fimmu.2021.618577 (PMC8009969; doi:10.3389/fimmu.2021.618577)
Supplement: Supplementary file 1 [file Data_Sheet_1.docx]

***Supplementary Material***

Dengue virus envelope protein domain III induces Nlrp3 inflammasome-dependent NETosis-mediated inflammation in mice

Te-Sheng Lien^1^, Der-Shan Sun^1,2^, Shih-Che Hung^2^, Wen-Sheng Wu^3,4^ and Hsin-Hou Chang^1,2 *^

^1^ Department of Molecular Biology and Human Genetics, Tzu-Chi University, Hualien 970, Taiwan.

^2^ Institute of Medical Sciences, Tzu-Chi University, Hualien 970, Taiwan.

^3^ Division of General Surgery, Department of Surgery, Hualien Tzu Chi Hospital, Buddhist Tzu Chi Medical Foundation, Hualien 970, Taiwan

^4^ School of Medicine, Tzu Chi University, Hualien 970, Taiwan

* To whom correspondence should be addressed: Hsin-Hou Chang, PhD. Department of Molecular Biology and Human Genetics, Tzu-Chi University, Hualien 970, Taiwan, ROC. Tel: 886-3-8565301 ext 2667. Fax: 886-3-8578386. E-mail:hhchang@mail.tcu.edu.tw

# Supplementary Figures 1-7

**Fig. S1**

**
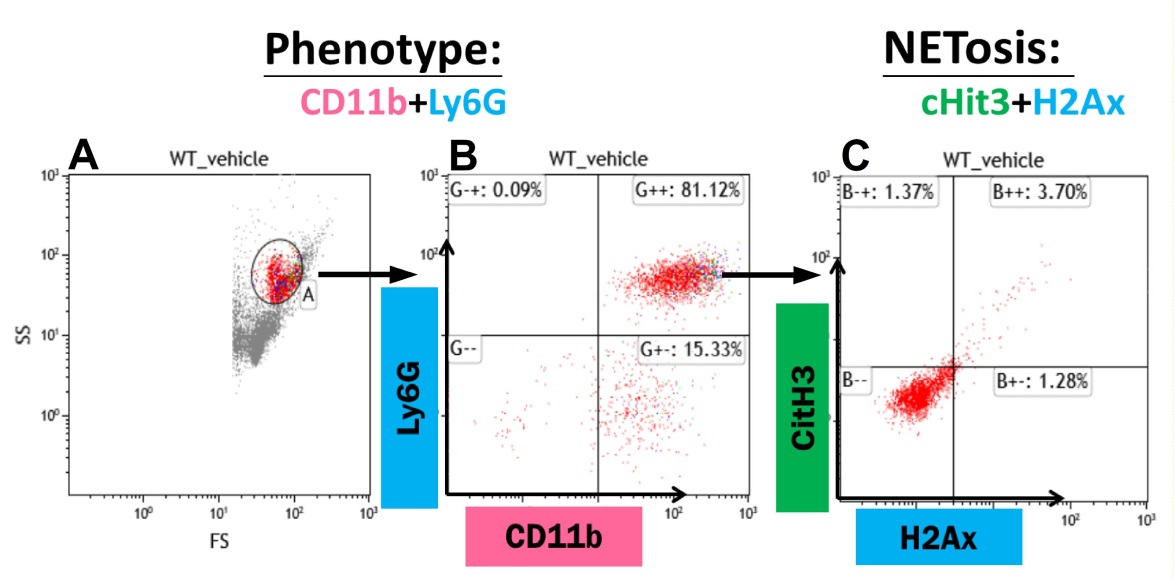
**

**Figure S1.** **The flow cytometry gating of rEIII-induced NETosis.** An example on the gating of flow cytometry analysis is showed. After cell size (forward scatter; FS) and granularity (side scatter; SS) gating (A), and CD11b and Ly6G staining (B), the neutrophil population could be obtained as the CD11b^+^Ly6G^+^ cells. The neutrophil population undergoing NETosis could be further characterized using cituninated-hsitone H3 (CitH3) and histone 2AX (H2AX) staining, and displayed as CitH3^+^H2AX^+^ double positive cells (C).

**Fig. S2**

**
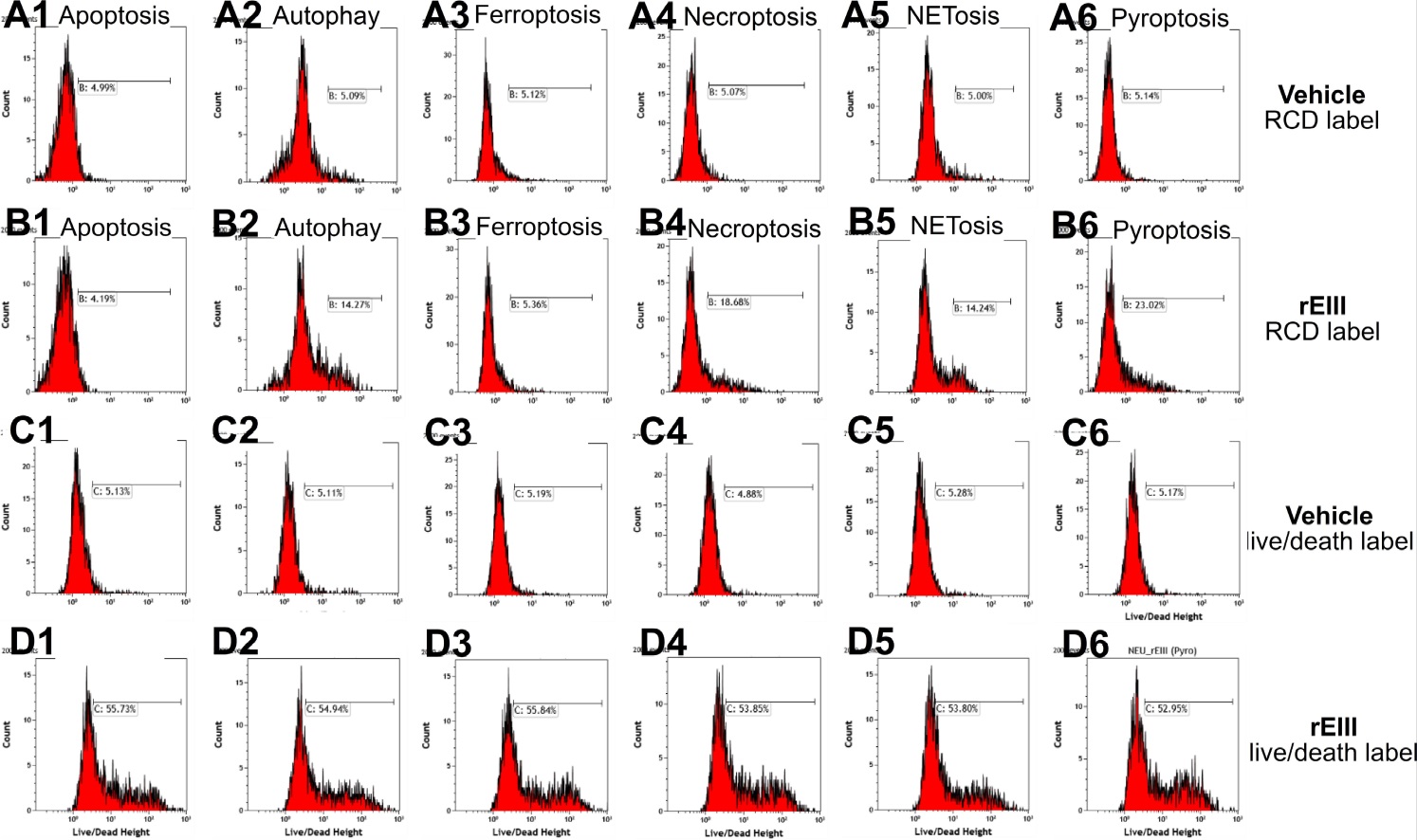
**

**Figure S2.** **Calculation of respective percentage of regulated cell death (RCD).** An example shows how respective RCD pathway percentages were determining by flow cytometry results. Because overlapping of detection wavelengths, it is not feasible to detect total 6 RCD pathways simultaneously in one cell-staining sample. Therefore, we performed double staining of respective RCD plus cell live and death statue staining (an internal control). For example, after neutrophil samples were treated with vehicle (A, C) or rEIII (B, D), these neutrophils were subjected to respective RCD (A, B) and cell-live/death status (C, D) staining. The respective increased cell death signal (e.g. apoptosis signal B1-A1; △apoptosis) was normalized (e.g. B1-A1/D1-C1; △ apoptosis/△total death cell) by increased death-cell population (e.g. D1-C1; △total death cell) of respective RCD staining. The sum of B1-A1/D1-C1, B2-A2/D2-C2, B3-A3/D3-C3, B4-A4/D4-C4, B5-A5/D5-C5 and B6-A6/D6-C6 was considered as 100%. As a result, the NETosis % was calculated as [B5-A5/D5-C5] / [(B1-A1/D1-C1) + (B2-A2/D2-C2) + (B3-A3/D3-C3) + (B4-A4/D4-C4) + (B5-A5/D5-C5) + (B6-A6/D6-C6)] × 100%, and is approximately 20%. Results showed in figure 3 were obtained through aforementioned formula using averaged results from triplicated samples of each group.

**Fig. S3**


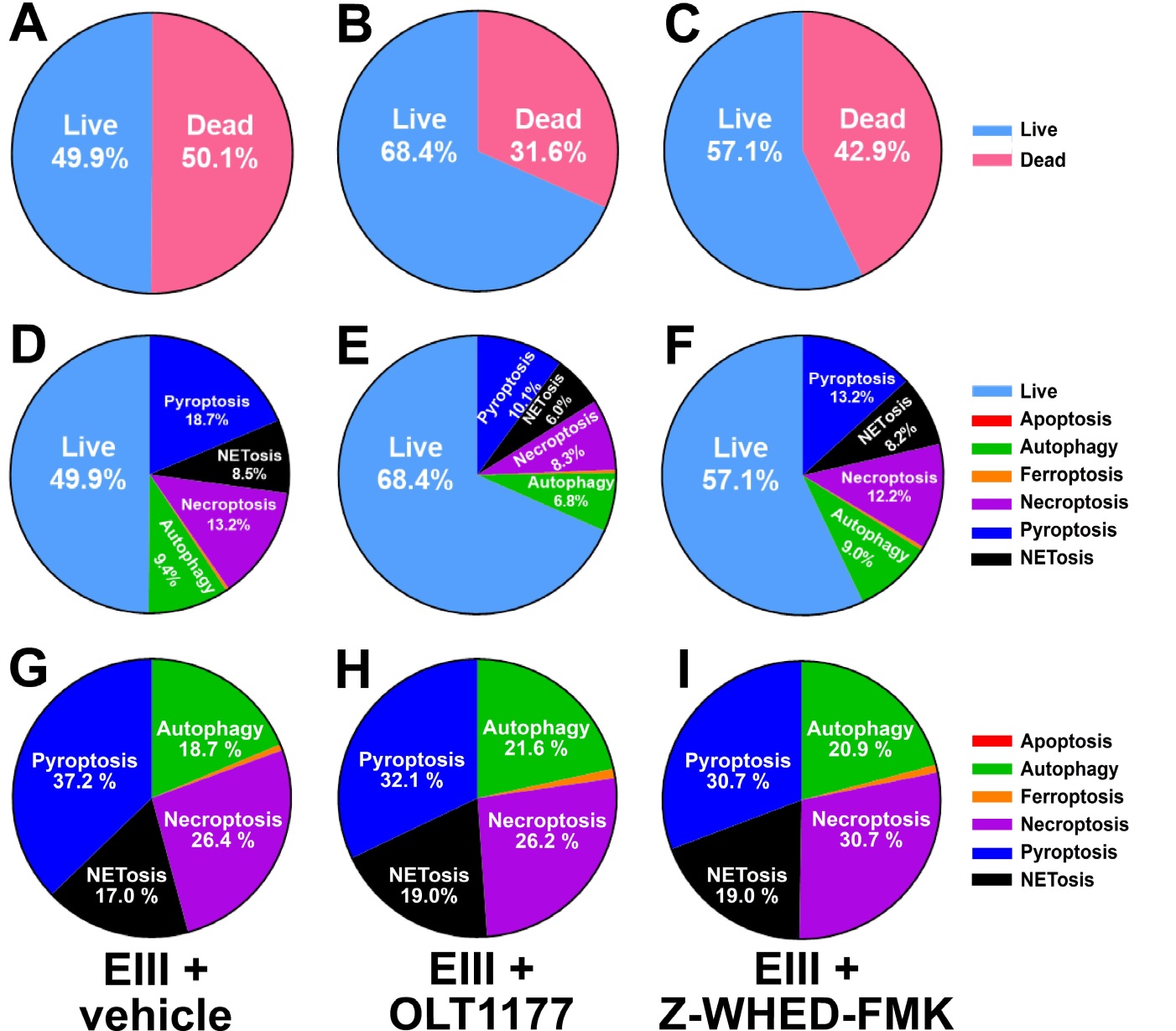


**Figure S3.** **The pie charts of OLT1177 and Z-WHED-FMK treatments on the influence of EIII-induced neutrophil RCDs.** Treatment with Nlrp3 inhibitor OLT1177 (10 μM) and caspase 1 inhibitor Z-WHED-FMK (10 μM) on the rescue of rEIII-induced neutrophil total cell death (A-C). Treatments with Nlrp3 inflammasome inhibitors OLT1177, and Z-WHED-FMK rescued pyroptosis, necroptosis, autophagy and NETosis, but not ferroptosis and apoptosis (D-F). If we normalize the respective RCD % by the population of death cells (dead cell population normalized to 100%), we found that OLT1177 and Z-WHED-FMK still display rescue effects on pyroptosis (G-I). n = 6.

**Fig. S4**

**
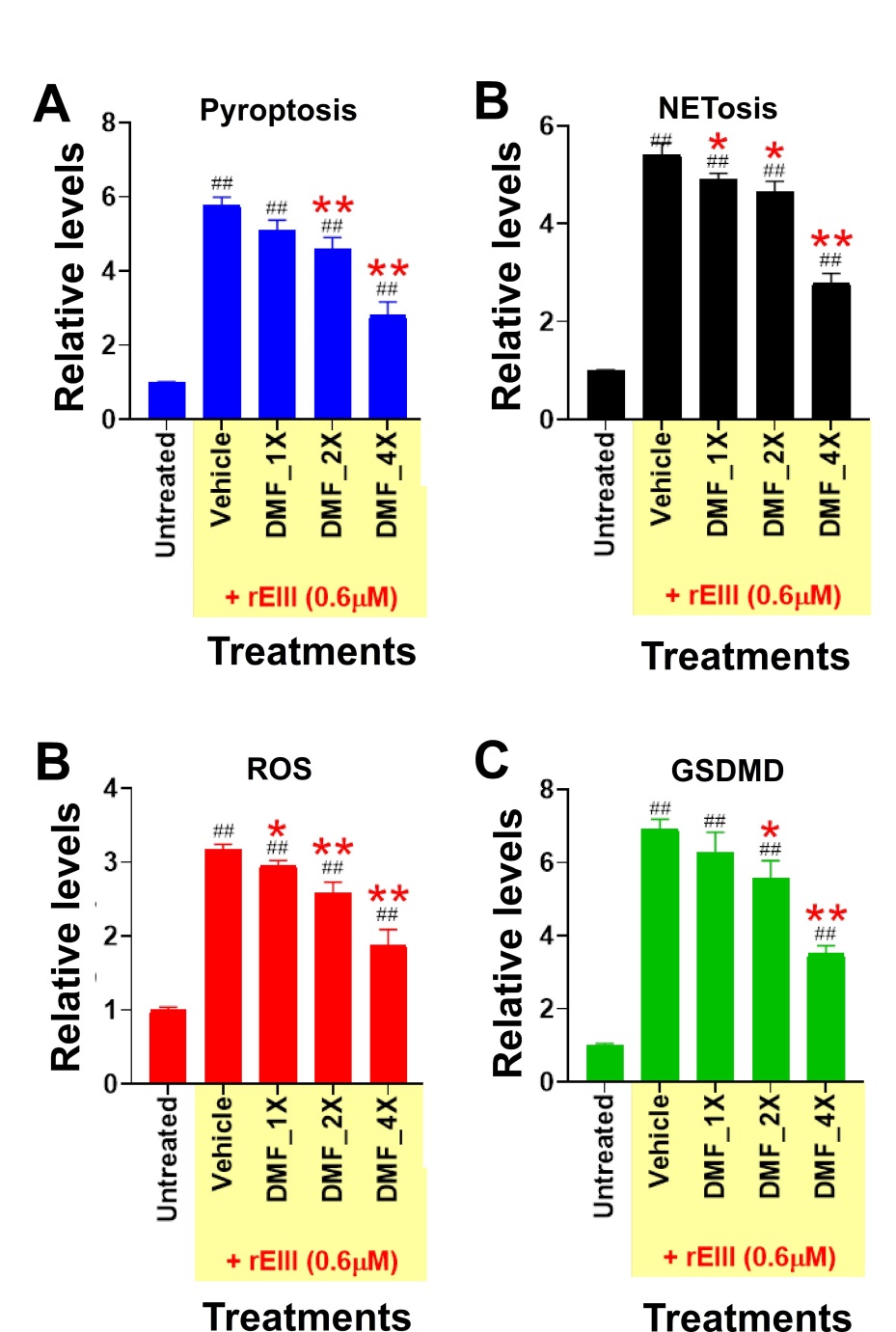
**

**Supplementary Figure 4. Treatments on the influence of EIII-induced mouse neutrophil pyroptosis, NETosis, cellular ROS, and surfaμce GSDMD levels.** Rescue of EIII (0.6 μM)-induced mouse neutrophil pyroptosis (A), NETosis (B), cellular ROS (C), and cell-surface GSDMD (D) levels by 1 h pretreatment of pyroptosis inhibitor DMF. 1 × DMF: 25 μM, 2 × DMF: 50 μM, 4 × DMF: 100 μM; n = 6, ## P < 0.01 vs. EIII-untreated groups; * P < 0.05, ** P < 0.01 vs. respective rEIII + vehicle groups.

**Fig. S5**

**Supplementary Figure 5. Suppression of rEIII-induced caspase-1 activation by treatments with pyroptosis and NETosis inhibitors.** Rescue of EIII (0.6 μM)-induced mouse neutrophil caspase-1 activation by 1 h pretreatments with pyroptosis inhibitors DMF (100 μM), OLT1177 (10 μM), Z-WHED-FMK (10 μM), and NETosis inhibitor GSK484 (2 nM). n = 6, * P < 0.05, ** P < 0.01 vs. respective rEIII + vehicle groups.

**Fig. S6**


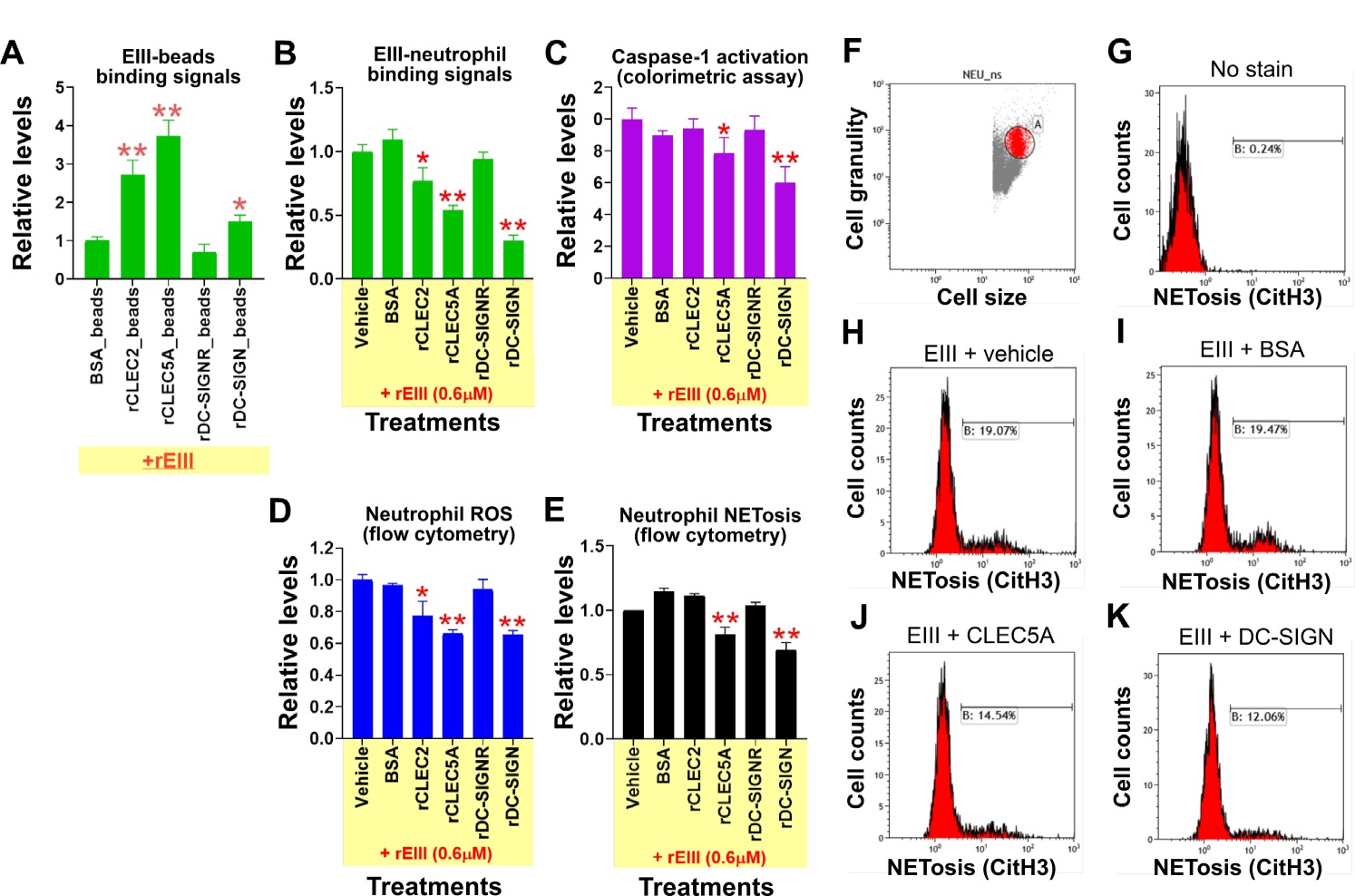


**Figure S6.** **CLEC5A and DC-SIGN are potential neutrophil cell surface targets of EIII.** Flow cytometry-based assays were performed. The biotin-labeled EIII binds to beads coated with various proteins (A), and neutrophils (B). EIII-induced neutrophil caspase-1 activation (C), ROS production (D) and EIII-induced neutrophil NETosis levels (E; F-K, flow cytometry gating), were competed by various soluble-form cellular receptor proteins, including recombinant CLEC2, CLEC5A, DC-SIGNR, DC-SIGN, and negative controls, vehicle and BSA (C, D; all proteins, 50 μg/mL). * *P* < 0.05, ** *P* < 0.01, significant higher vs. BSA-coated beads groups (A); * *P* < 0.05, ** *P* < 0.01, significant decrease vs. vehicle control groups (B, C); ** *P* < 0.01, vs. vehicle control groups, ## *P* < 0.01, vs. control Ig groups (D); * *P* < 0.05, vs. vehicle control groups (E).

**Fig. S7**

**
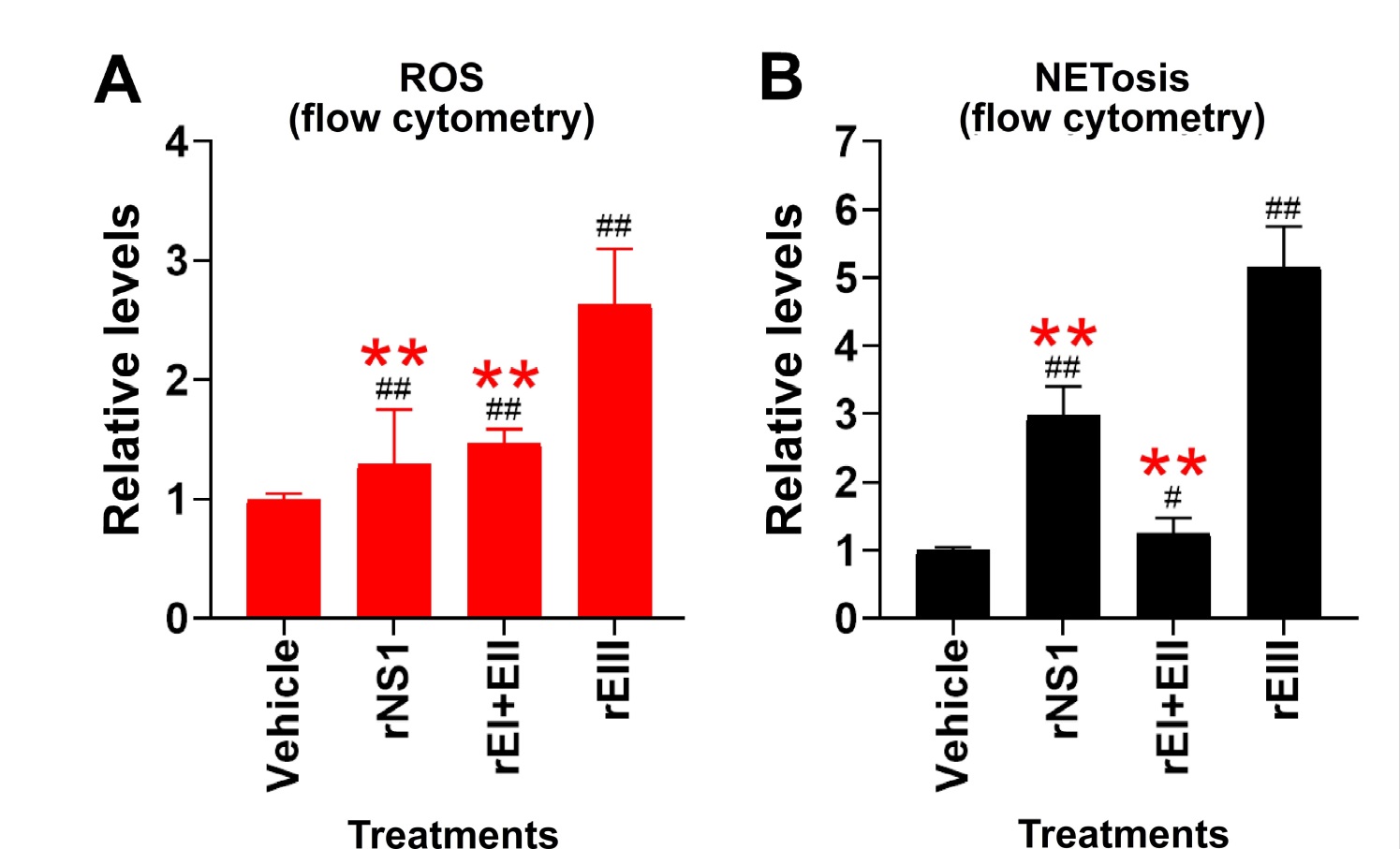
**

**Supplementary Figure 7. Treatments with DENV rNS1 and rEIII increase mouse neutrophil ROS and NETosis levels.** Flow cytometry analysis on the percentage of DENV rNS1 (0.6 μM), rEI+EII (DENV envelop protein domain I + domain II; used as a control group; 0.6 μM) and rEIII (0.6 μM)-induced ROS (A) and NETosis (B) in total population. Dosage used, rNS1, 0.6 μM, rEIII, 0.6 μM, both are adjusted approximately reached to the clinically detected levels. n = 6. # P < 0.05, ## *P* < 0.01, vs. respective vehicle groups; ** *P* < 0.01, vs. respective rEIII groups.
